# Increase of IFN-γ and TNF-α production in CD107a + NK-92 cells co-cultured with cervical cancer cell lines pre-treated with the HO-1 inhibitor

**DOI:** 10.1186/s12935-014-0100-1

**Published:** 2014-10-01

**Authors:** Paulina Gómez-Lomelí, Alejandro Bravo-Cuellar, Georgina Hernández-Flores, Luis Felipe Jave-Suárez, Adriana Aguilar-Lemarroy, José Manuel Lerma-Díaz, Jorge Ramiro Domínguez-Rodríguez, Karina Sánchez-Reyes, Pablo Cesar Ortiz-Lazareno

**Affiliations:** División de Inmunología, Centro de Investigación Biomédica de Occidente (CIBO), Instituto Mexicano del Seguro Social (IMSS), Sierra Mojada 800, Col. Independencia, 44340 Guadalajara, Jalisco Mexico; Departamento de Ciencias de la Salud, Centro Universitario de los Altos, Universidad de Guadalajara (UdeG), Tepatitlán de Morelos, Jalisco Mexico; Departamento de Farmacobiología, Centro Universitario de Ciencias Exactas e Ingeniería, UdeG, Guadalajara, Jalisco Mexico; Programa de Doctorado en Ciencias Biomédicas Orientación Inmunología, Centro Universitario de Ciencias de la Salud (CUCS), UdeG, Guadalajara, Jalisco Mexico

**Keywords:** Heme oxygenase 1, NK cells, Cervical cancer cells, IFN-γ, TNF-α, NKp30, NKG2D

## Abstract

**Background:**

Natural killer (NK) cells eliminate virus-infected and tumor cells through the release of perforins and granzymes; they also produce Interferon gamma (IFN-γ) and Tumor necrosis factor alpha (TNF-α), which induce apoptosis in target cells. Many tumors express Heme oxygenase 1 (HO-1), and this expression has been associated with avoiding immunosuppression and apoptosis. In this work, HO-1+ Cervical cancer cell (CCC) lines were pre-treated with HO-1 inhibitor and we assessed whether this inhibition enhanced the sensitivity of CCC to NK cell activity.

**Methods:**

We assessed the expression of HO-1 in HeLa, SiHa, and C-33A CCC by Flow cytometry (FC). CCC were pre-treated with SnPP or ZnPP HO-1 inhibitors. After that, NK-92 cells were co-cultured with HeLa, SiHa, and C-33A CCC pre-treated or not with HO-1 inhibitors, and the expression of IFN-γ, TNF-α, CD107a, Granzyme B, NKp44, NKp46, NKp30, and NKG2D was evaluated by FC.

**Results:**

CCC lines HeLa, SiHa, and C-33A expressed HO-1. Inhibition of HO-1 in these cells increased the expression of IFN-γ and TNF-α in CD107a + NK-92 cells. We observed a reduction in the expression of NKG2D, NKp46, and NKp30 in NK cells co-cultured with HeLa and SiHa cells, and when HeLa and SiHa cells were pre-treated with the HO-1 inhibitors, the expression of NKG2D and NKp30 in NK cells was restored. We observed a similar effect in NK cells co-cultured with C-33A cells in NKp30 expression.

**Conclusion:**

Inhibition of HO-1 in CCC induces an increase in IFN-γ and TNF-α production in CD107a + NK-92 cells and restores NKG2D, NKp46 and NKp30 downmodulation in NK cells.

## Background

Cervical cancer is the third most common cancer among women worldwide [1] The main risk factor for cervical cancer is infection with the Human papillomavirus (HPV) [2,3]. HPV types 16 and 18 are responsible for 70% of cases [4]. The immune system is responsible for eliminating tumor cells and infected cells with microorganisms or foreign antigens. Natural killer (NK) cells are the major cells responsible for tumor removal and elimination of infected cells [5]. NK cells constitute between 2 and 18% of total lymphocytes in peripheral blood and are distributed in lymphoid as well as in non-lymphoid organs. NK cells respond to cellular signals triggered by receptor activation or inhibition; once they interact with their specific target, if these signals trigger NK cell activation, the release of perforins and granzymes induces cellular lysis in target cells [6]. Another important function of NK cells is the production of cytokines, such as Interferon gamma (IFN-γ) and Tumor necrosis factor alpha (TNF-α); these cytokines also act as promoter agents of apoptosis in tumor cells, or they recruit and activate other cells of the immune response, such as monocytes/macrophages, dendritic cells, T cells, and B cells [7,8]. The production of these cytokines by NK cells promotes innate and adaptive immunity [9]. The major activating receptors in NK cells are NGK2D and Natural cytotoxicity receptors (NCR), that is, NKp30, NKp44, and NKp46 [10]. These activating receptors recognize ligands in tumor or infected cells [11]. These ligands for activating receptors are stress-inducible molecules and include UL16-binding proteins (ULBP) and (MHC) class I-related chain A/B (MICA/B) recognized by NKG2D [12,13]. Other ligands include the poliovirus to DNAM-1, the HLA-B-associated transcript 3 (BAT-3), and the B7-H6 molecule, both recognized by NKp30 [14,15]. These ligands are absent in normal cells and their expression is increased in cancer cells. However, interaction among immune cells, tumor cells, and neighboring cells is very complex. Cells are influenced by the surrounding microenvironment and this process involves a selection mechanism of tumor cells, which initiate a number of signaling mechanisms to evade the immune response, as described in the hypothesis of immunoediting [16]. Under certain circumstances, this interaction culminates in the eradication of tumor cells, as occurs the majority of times, or instead, in suppression of the immune response and tumor formation. Within this context, tumor cells developed various escape mechanisms to avoid NK-mediated killing; tumor cells produced cytokines, growth factors, and enzymes that induced suppression of cells of the immune response [17,18]. Heme oxygenase 1 (HO-1) is the rate-limiting enzyme in heme catabolism and leads to three products: biliverdin; free iron, and carbon monoxide [19]. It plays an important role in the modulation of inflammation, blocking the apoptotic process and antioxidant defense in the presence of any damage [20,21]. This enzyme is overexpressed in pancreatic, colon, and lung cancer, in which it can promote tumor cell proliferation and resistance to tumor therapy [22,23]. Induction of HO-1 in tumor cells undergoing any stressor agent increases their resistance to apoptosis [24-26]. Likewise, inhibition of this enzyme leads to the reduction of tumor growth and an increase in sensitivity to chemotherapy [19,27]. In this study, we evaluated HO-1 expression in Cervical cancer cells (CCC) and whether HO-1 inhibition enhanced the sensitivity of CCC to NK cells.

## Results

### Cervical cancer cell lines express HO-1

**Figure 1:**
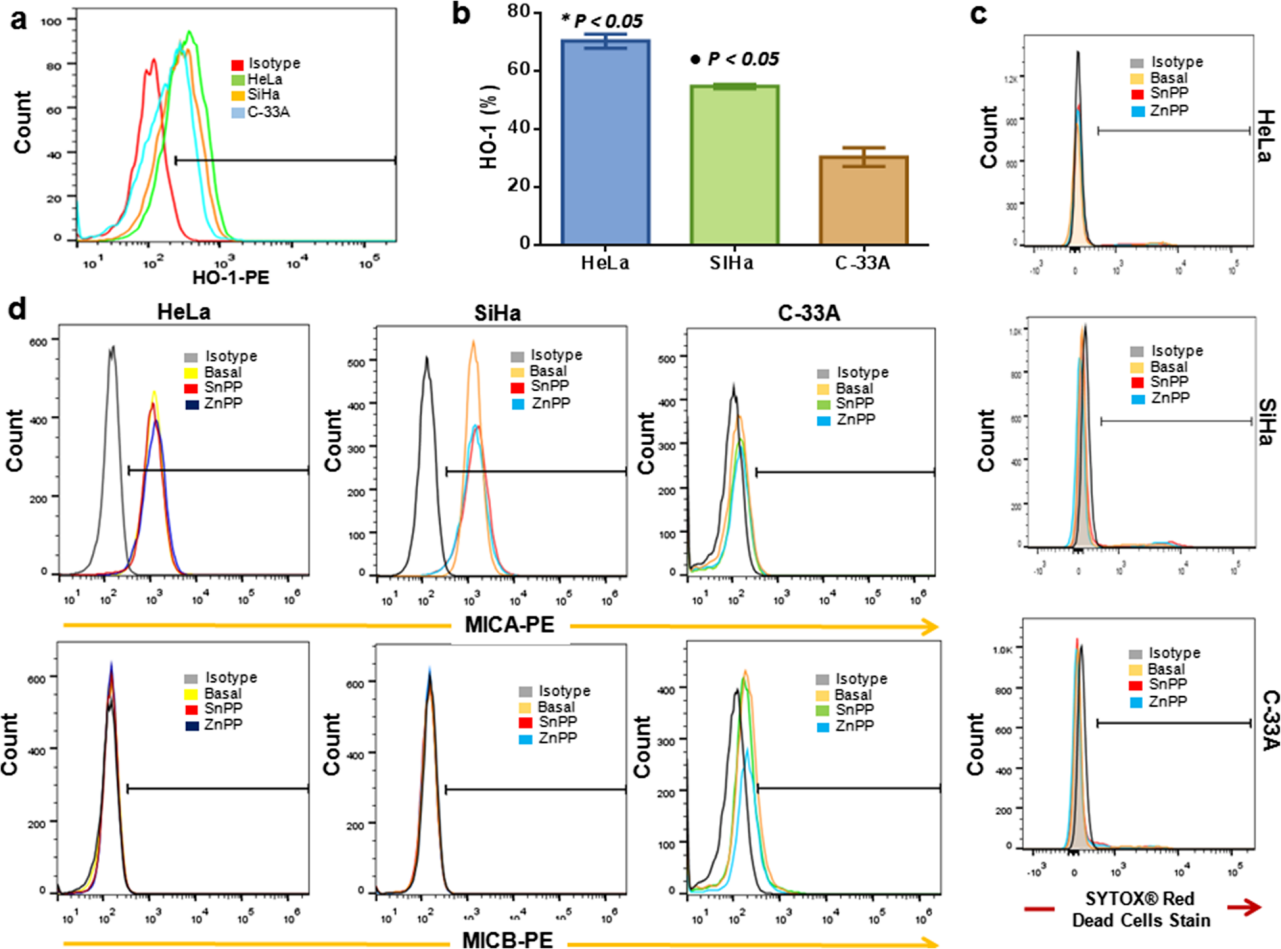
**Expression of Heme oxygenase 1 (HO-1) in different Cervical cancer cell (CCC) lines.** Expression of HO-1 in HeLa, SiHa, and C-33A cells was detected by indirect staining protocol using a PE-conjugated anti-mouse secondary antibody after incubation with mouse anti-HO-1 primary antibody. Results represent the mean ± Standard deviation (SD) of three independent experiments carried out in triplicate. A representative experiment of HO-1 expression in HeLa, SiHa, and C-33A cells is shown **(a)**. Percentage of HO-1 expression in HeLa, SiHa, and C-33A cells **(b)**. After treatment with HO-1 inhibitors, viability in HeLa, SiHa, and C-33A cells was evaluated with Sytox by Flow cytometry (FC) **(c)**. Expression of MICA and MICB in HeLa, SiHa, and C-33A cells treated or not with SnPP (25 μM) or ZnPP (1 μM), (HO-1 inhibitors) **(d)**. *P <0.05 HeLa, SiHa vs. C-33A cells.

We evaluated HO-1 expression in HeLa (HPV 18+), SiHa (HPV 16+), and C-33A (HPV–) CCC (Figures 1a and b). We can observe that the HeLa cell is the cancer cell line that expressed the highest percentage of cells positive to HO-1 (70.2% ± 4.9%) in comparison with SiHa and C-33A CCC (54.6% ± 1.5% and 30.3% ± 6.5%, respectively) (*p* <0.01). Additionally, we determined the geometric Mean fluorescence intensity (MFI) in each cancer cell line. HeLa, SiHa, and C-33A lines have similar MFI, and we did not observe a difference for HO-1 MFI among the three CCC, suggesting that the difference it is not in the intensity of expression, but rather in the number of cells positive to HO-1. Likewise, we evaluated viability in CCC treated with SnPP (25 μM) and ZnPP (1 μM) HO-1 inhibitors and observed that these inhibitors did not affect the viability in these cells (Figure 1c). In addition, we evaluated whether HO-1 inhibitors affect the expression of NK cell ligands, such as MICA and MICB. HeLa and SiHa cells express MICA, but not MICB, while C-33A expresses MICB, but not MICA, and we did not observe a change in MICA or MICB expression when cells were treated with SnPP or ZnPP inhibitors (Figure 1d). HO-1 inhibitors did not affect MICA and MICB receptors.

### CD107a expression in NK-92 cells co-cultured either with cervical cancer cells pre-treated or not with the SnPP, HO-1 inhibitor

**Figure 2:**
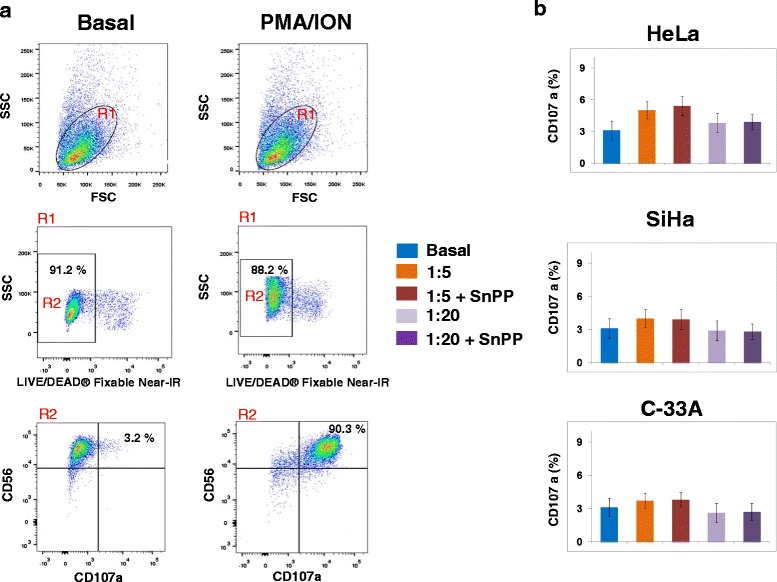
**Expression of CD107a in NK-92 cells co-cultured with Cervical cancer cells (CCC) pre-treated or not with Heme oxygenase 1 (HO-1) inhibitor.** CCC were pre-treated with the HO-1 inhibitor (SnPP) for 48 h; afterward, NK-92 cells were co-cultured with HeLa, SiHa, and C-33A CCC for 4 h. Then, the expression of CD107a was analyzed by Flow cytometry (FC). Expression of CD107a in NK-92 cells and in PMA/Ionomycin groups **(a)**. NK-92/CCC co-culture **(b)**. PMA/Ionomycin was used as positive control. These data are representative of three independent experiments.

We evaluated the expression of CD107a in NK-92 cells co-cultured with HeLa, SiHa, and C-33A CCC pre-treated or not with HO-1 inhibitor (SnPP) (Figure 2). In Figure 2a, we can observe the baseline expression of CD107a in NK-92 cells and the positive-control PMA/Ionomycin increase of this expression. We did not observe differences in NK-92 cells co-cultured with HeLa cells pre-treated or not with HO-1 inhibitor in all target effector ratios (T:E) 1:5 and 1:20 (Figure 2b). In NK-92 cells co-cultured with SiHa and C-33A CCC, we observed similar behavior to that observed for HeLa; there were no significant differences between the different T:E ranges between pre-treated cells or not treated with the HO-1 inhibitor. When we analyzed MFI for CD107a, there was no difference in any of the experimental groups; as we expected, only the positive control group (PMA + Ionomycin) increased expression and MFI of CD107a in NK-92 cells.

### Increase of IFN-γ and TNF-α production in NK-92 cells positive to CD107a co-cultured with cervical cancer cell lines pre-treated with the HO-1 inhibitor

**Figure 3:**
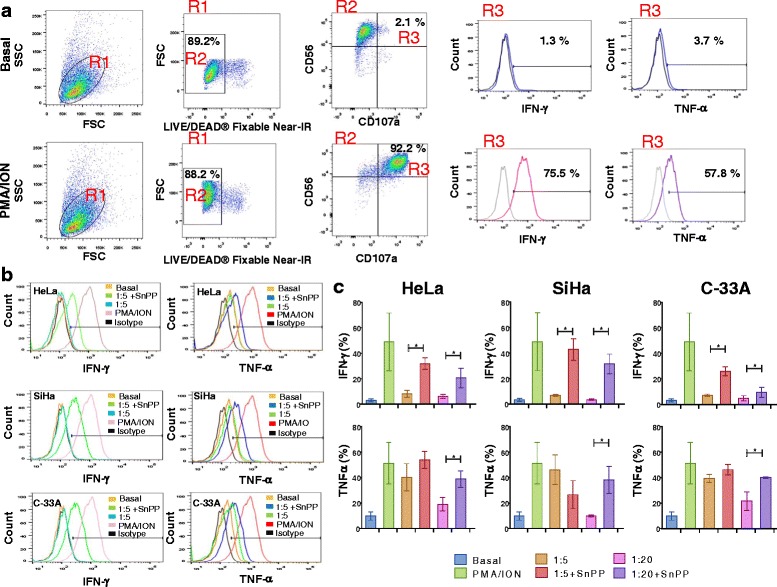
**Increase of Interferon gamma (IFN-γ** **) and Tumor necrosis factor alpha (TNF-α** **) in CD107a + NK-92 cells co-cultured with Cervical cancer cell (CCC) lines pre-treated with Heme oxygenase 1 (HO-1) inhibitor.** CCC were pre-treated with HO-1 inhibitor (SnPP) for 48 h; after that, NK-92 cells were co-cultured with HeLa, SiHa, and C-33A CCC for 4 h. Then, the expression of IFN-γ and TNF-α was analyzed in CD107a + NK-92 cells by Flow cytometry (FC). Expression of IFN-γ and TNF-α in CD107a + NK-92 cells without co-culture and in NK-92 cells stimulated with PMA/Ionomycin (positive control), gray profiles represent isotype controls **(a)**. A representative experimental of IFN-γ and TNF-α production in CD107a + Natural killer (NK)-92/CCC co-culture **(b)**. The percentage of IFN-γ and TNF-α production in CD107a + NK-92/CCC co-culture **(c)**. Bars represent the mean ± Standard deviation (SD) of three independent experiments carried out in triplicate. *P <0.05 1:5 + SnPP or 1:20 + SnPP vs. 1:5 or 1:20 in HeLa, SiHa, and C-33A. Mann–Whitney *U* test.

In Figure 3a, we observe the baseline expression of INF-γ and TNF-α in CD107a + NK-92 cells (Basal group) and in the positive control group (PMA/Ionomycin) that induced degranulation in NK-92 cells. In Figures 3b and c, we can observe that in NK-92 cells co-cultured with HeLa cells pre-treated with HO-1 inhibitor SnPP, there is an increase of IFN-γ production (*p* <0.05) in comparison with HeLa cells without pre-treatment with the HO-1 inhibitor, in which the highest IFN-γ expression fell within the range of 1:5. When NK-92 cells were co-cultured with SiHa cells, we observed a similar effect to that observed in HeLa cells. In this case, the increase in IFN-γ expression was observed when SiHa cells were pre-treated with SnPP (the HO-1 inhibitor) (*p* <0.05). When NK-92 cells were co-cultured with C-33A pre-treated with HO-1 inhibitor, we can observe a significant increase in the production of IFN-γ in comparison with C-33A without pre-treatment with the HO-1 inhibitor (*p* <0.05). In general, there was an increase in the production of IFN-γ in CD107a + NK-92 cells co-cultured with HeLa, SiHa, and C-33A pre-treated with the HO-1 inhibitor (SnPP). In the same Figure 3b and c, we can observe TNF-α production in CD107a + NK-92 cells co-cultured with HeLa, SiHa, and C-33A pre-treated or not with the HO-1 inhibitor. In the co-culture with HeLa cells, when tumor cells were pre-treated with the HO-1 inhibitor, TNF-α production was significant in NK-92 cells (*p* <0.05). We observed a similar difference in the SiHa co-culture. Production of TNF-α in NK-92 cells co-cultured with C-33A pre-treated with the HO-1 inhibitor is significantly increased within the 1:20 range (*p* <0.05).

### Granzyme B expression in CD107a + NK-92 cells co-cultured with cervical cancer cell lines pre-treated with the HO-1 inhibitor

**Figure 4:**
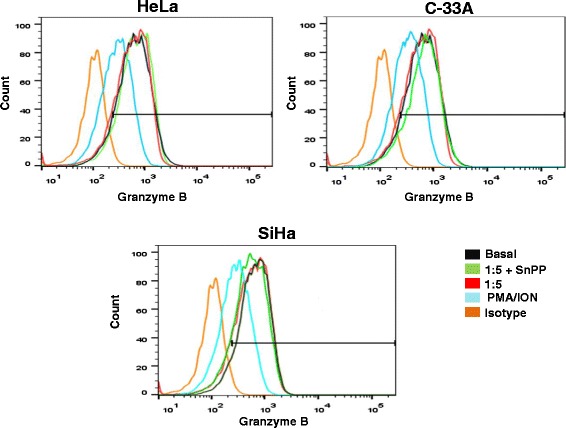
**Expression of granzyme B in CD107a + NK92 cells co-cultured with Cervical cancer cell (CCC) lines pre-treated or not with the Heme oxygenase 1 (HO-1) inhibitor.** CCC were pre-treated with the HO-1 inhibitor (SnPP) for 48 h; afterward, NK-92 cells were co-cultured with CCC for 4 h. Then, the expression of granzyme B was analyzed in CD107a + NK-92 cells co-cultured with HeLA, SiHa, and C-33A pre-treated or not with the SnPP by Flow cytometry (FC). A representative experiment is shown.

We evaluated granzyme B expression in CD107a + NK-92 cells co-cultured with HeLa, SiHa, and C-33A CCC pre-treated or not with the HO-1 inhibitor (Figure 4). We did not observe differences in granzyme B release in any T:E ratios 1:5 and (1:20 and 1:40, data not shown) in NK-92 cells co-cultured with HeLa, SiHa, and C-33A CCC pre-treated or not with the HO-1 inhibitor. It is important to stress that PMA/Ionomycin induces the release of granzyme B and correlates with the association between CD107a increase and granzyme B release.

### Supernatant of cervical cancer cell lines HeLa, SiHa, and C-33A induces downmodulation of NCR and NKG2D receptors in NK cells

**Figure 5:**
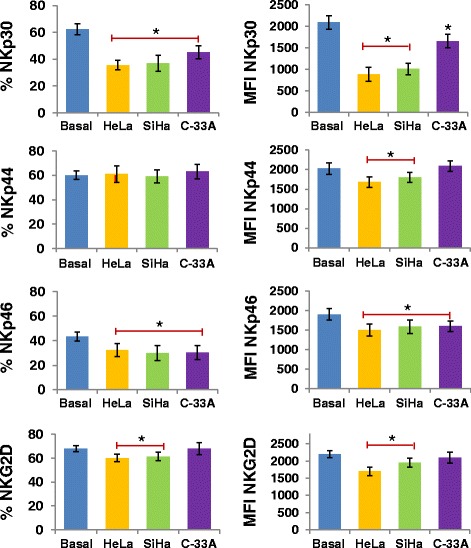
**Downmodulation of NCR and NKG2D in NK-92 cells treated with the supernatant of HeLa, SiHa, and C-33A Cervical cancer cells (CCC).** Supernatant of CCC was added to a final concentration of 30% in NK-92 cells. Then, the cells were incubated for 24 h at 37°C in a humid atmosphere containing 5% CO_2_ and 95% air in RPMI-S medium. Afterward, the expression of NCR or NKG2D was analyzed by Flow cytometry (FC). Values of percentage of expression and geometric Mean fluorescence intensity (MFI) for NKp30, NKp44, NKp46, and NKG2D in NK-92 cells treated or not with the supernatant of HeLa, SiHa, and C-33A CCC. Results are represented as the mean ± Standard deviation (SD) of three independent experiments performed in triplicate. *P <0.05 HeLa, SiHa, and C-33A vs. basal group, or HeLa, SiHa vs. basal group, Mann–Whitney *U* test.

**Figure 6:**
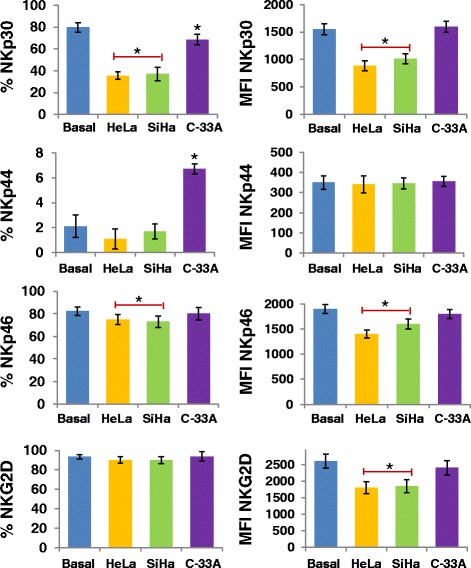
**Downmodulation of NCR and NKG2D in NK cells treated with the supernatant of HeLa, SiHa, and C-33A Cervical cancer cells (CCC).** Supernatant of CCC was added to a final concentration of 30% in primary NK cells of healthy donors. Then, the cells were incubated for 24 h at 37°C in a humid atmosphere containing 5% CO_2_ and 95% air in RPMI-S medium. Afterward, the expression of NCR or NKG2D was analyzed by Flow cytometry (FC). Values of percentage of expression and geometric Mean fluorescence intensity (MFI) for NKp30, NKp44, NKp46, and NKG2D in NK cells treated or not with the supernatant of HeLa, SiHa, and C-33A CCC. Results are represented as the mean ± Standard deviation (SD) of three independent experiments performed in triplicate. *P <0.05 HeLa, SiHa, and C-33A vs. basal group, or HeLa, SiHa vs. basal group, Mann–Whitney *U* test.

We investigated whether the supernatant of CCC HeLa, SiHa, and C-33A induces downmodulation of NKp30, NKp44, NKp46 and NKG2D in NK-92 cell line and NK cells of healthy donors. We observed that in both cells, HeLa and SiHa cancer cells induce downmodulation of NKp30, NKp46, and NKG2D in NK cells, and in C-33A cells only NKp30 and NKp46 (*p* <0.05 Figures 5 and 6). Likewise, in NK-92 cells we observed downmodulation of NKp44 when the cells were treated with the supernatant of HeLa and SiHa (*p* <0.05).

### The HO-1 inhibitor restores downmodulation in NCR and NKG2D expression in NK-92 cells co-cultured with cervical cancer cell lines

**Figure 7:**
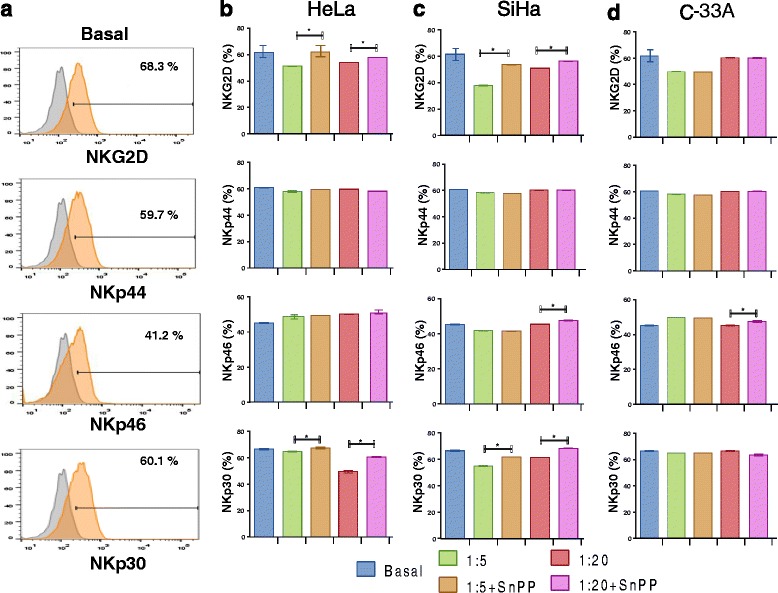
**Downmodulation of NKG2D, NKp46, and NKp30 in NK-92 cells co-cultured with Cervical cancer cell (CCC) lines.** CCC were pre-treated with the Heme oxygenase 1 (HO-1) inhibitor (SnPP) for 48 h; after that, NK-92 cells were co-cultured with HeLa, SiHa, and C-33A CCC for 4 h. Then, the expression of NKp30, NKp44, NKp46, and NKG2D was analyzed in NK-92 cells by Flow cytometry (FC). Expression of NKp30, NKp44, NKp46, and NKG2D in NK-92 (Basal group) **(a)**. Expression of NKp30, NKp44, NKp46, and NKG2D in NK92/HeLa cell co-culture **(b)**. Expression of NKp30, NKp44, NKp46, and NKG2D in NK92/SiHa cell co-cultures **(c)**. Expression of NKp30, NKp44, NKp46, and NKG2D in NK92/C-33A cell co-cultures **(d)**. Bars represent the mean ± Standard deviation (SD). Results are represented as the mean ± Standard deviation (SD) of three independent experiments performed in triplicate. *P <0.05 1:5 + SnPP or 1:20 + SnPP vs. 1:5 or 1:20 in NKG2D, NKp46, and NKP30 in HeLa, SiHa, or C-33A. Mann–Whitney *U* test.

We evaluated the expression of NKp30, NKp44, NKp46, and NKG2D in NK-92 cells co-cultured with HeLa, SiHa, and C-33A cancer cells pre-treated or not with the HO-1 inhibitors. We can observe that in NK-92 cells co-cultured with HeLa cells pre-treated with SnPP (the HO-1 inhibitor), there is an increase in NKG2D, and NKp30 expression within the different T:E ratios (*p* <0.05) (Figure 7) in comparison with HeLa cells without pre-treatment with the HO-1 inhibitor. When NK-92 cells were co-cultured with SiHa cells, we observed a similar effect to that observed with HeLa cells, and an increase in NKG2D, NKp46, and NKp30 receptor when SiHa cells were pre-treated with SnPP (*p* <0.05), in comparison with SiHa cells without pre-treatment. When NK-92 cells were co-cultured with C-33A pre-treated with the HO-1 inhibitor, we are able to observe a significant increase in the expression of NKp46 within the 1:20 ratio (*p* <0.05) in comparison with cells without pre-treatment. We observed similar and comparable results when we used the ZnPP inhibitor (data not shown). Similarly, we performed transwell assays to evaluated change in NCR and NKG2D expression in NK-92 and NK cells of healthy donors. We can observe, in Table 1, that the greatest change observed was in NKp30 and NKG2D receptors in HeLa and SiHa cells, and that when NK-92 cells were treated with HO-1 inhibitors (SnPP and ZnPP), this downmodulation is partially recovered (*p* <0.05). We observed similar results with NK primary cells of healthy donors (data not shown).

**Table 1:** **Downmodulation of NCR and NKG2D in NK-92 cells co-cultured with Cervical cancer cell (CCC) lines**

	**Hela**	**SiHa**	**C-33A**
NKG2D			
Basal	2235	2235	2235
Without HO-1 Inhibitor	1690	1830	2110
SnPP	2145*	2050*	2198
ZnPP	2189*	2012*	2241
NKp30			
Basal	2127	2127	2127
Without HO-1 Inhibitor	888	1609	1658
SnPP*	1848	1949	2087
ZnPP*	1929	1893	1989
NKp44			
Basal	2025	2025	2025
Without HO-1 Inhibitor	1684	1801	2090
SnPP	2110*	2087*	2089
ZnPP	1989*	2046*	2105
NKp46			
Basal	1900	1900	1900
Without HO-1 Inhibitor	1500	1588	1610
SnPP*	1925	2043	1892
ZnPP*	1875	1984	1931

CCC HeLa, SiHa, and C-33A were pre-treated with SnPP or ZnPP HO-1 inhibitors. Afterward, transwell assays were performed between NK-92 cells and HeLa, SiHa, and C-33A. Subsequently, the geometric Mean fluorescence intensity (MIF) of NKG2D, NKp30, NKp44, and NKp46 were determined. The Standard deviation (SD) of MIF in all groups did not exceed 135. *P <0.05 SnPP- or ZnPP-treated CCC vs. HeLa, SiHa, and C-33A without treatment.

## Discussion

Tumor cells acquire different capabilities that allow them to grow uncontrollably and even to evade the immune response. Among these acquired skills, we find the expression of certain cytokines, chemokines, and enzymes that are not normally expressed and that can facilitate an evasion of this type, such as Transforming growth factor beta (TGF-β), Interleukin (IL)-10, Prostaglandin E2 (PGE2), and indoleamine 2,3-dioxygenase [28]. In this regard, HO-1 possesses an important immune protective effect [29]. This enzyme is vital for the removal of heme, a potent pro-oxidant and inflammatory agent. HO-1 induces heme degradation and produces three metabolites: CO; ferrous iron, and biliverdin, all these metabolites with an immune protective effect [30]. We show that CCC HeLa, SiHa, and C-33A express HO-1; while each cell has a different percentage of expression of HO-1, all possess a similar amount of HO-1 (MFI were similar in HeLa, SiHa, and C-33A cells). The expression of HO-1 has been associated with anti-apoptotic and anti-inflammatory effects [31]. In this respect, it was published that HO-1 can induce the production of IL-10 and that this promotes suppression of the immune response [32,33]. Overexpression of HO-1 has been reported in many cancer types, such as colorectal, pancreatic, and prostate [34]. Likewise, it was published that HO-1 is involved in macrophage polarization toward an M2 phenotype [35], and that HO-1+ macrophages induce suppression of the immune response [36]. In this regard, it was also observed that HO-1 expressed by dendritic cells favors the emergence of CD4 + CD25+ regulatory T cells and inhibits the proliferation of T cells [37]. NK cells comprise the major cells involved in early responses against infected and tumor cells [38]. CD107a is associated with lysosomal membranes and has been associated with the degranulation of NK cells and CD8+ T cells, having been adopted as a marker of activation due to the association between the increased expression and increased cytotoxicity of their target cells [39,40]. In our studies, we did not observe a change in the expression of CD107a in NK cells co-cultured with HeLa, SiHa, or C-33A CCC pre-treated or not with the HO-1 inhibitor (SnPP), although we did observe an increase in the production of TNF-α and IFN-γ in CD107a + NK-92 cells co-cultured with cancer cells pre-treated with the HO-1 inhibitor. In this regard, in our study, when NK cells were co-cultured with HeLa, SiHa, and C-33A CCC pre-treated or not with the HO-1 inhibitor (SnPP), we did not observe a difference in the expression of CD107a and granzyme B in NK-92 cells. Although HeLa, SiHa, and C-33A cells express HO-1, this enzyme does not participate in the blocking of NK cell degranulation, because pre-treatment with the inhibitor of the enzyme does not induce changes in CD107a expression. Suppression of cytotoxicity in NK cells is associated with an important reduction in CD107a surface expression and suppression of granzyme B release [41]. It is likely that HO-1 does not interfere or intervene with the process of NK cell degranulation and cytotoxicity. It is noteworthy that CCC HeLa, SiHa, and C-33A, through other molecules, could interfere with the degranulation and cytotoxicity process in NK cells, as has been demonstrated with PGE_2_ [10], although we observed a significant increase of CD107a expression and cytotoxicity in K562 using NK-92 cells, and CD107a upregulation correlates with lysis of the target cells (data not shown). The cytotoxic action of NK cells comprises a series of steps involving combining the adhesion, activation, and secretion of lytic granules and other molecules, and NK cytotoxicity results are dependent on several changes that occur, such as phenotypical, functional, and molecular. While CD107a expression may not necessarily correlate with NK cytotoxicity, NK cells can also induce cell death by means of other mechanisms, such as FasL and TRAIL [42,43]. Previous studies demonstrated that products of the heme catabolism, such as CO, induced, in T cells, a reduction in IL-2 secretion and proliferation of the cells via inhibition of the ERK MAPK pathway [44]. Similarly, the use the metabolites induced by the action of HO-1 on a heme group, such as CO, ferritin, or bilirubin alone or in combination, induces a decrease in the production of TNF-α, IL-1, IL-6, and IFN-γ, and instead promotes IL-10 production [45,46]. It was in our interest to investigate whether inhibition of HO-1 in tumor cells increased the production of IFN-γ and TNF-α in the population of CD107a + NK-92 cells in our study. We observed that inhibition of the enzyme HO-1 increased IFN-γ and TNF-α expression in NK-92 cells co-cultured with HeLa, SiHa, and C-33A cells pre-treated with the HO-1 inhibitor. We observed, in SiHa (HPV 16+) cells pre-treated with the HO-1 inhibitor, a strong increase in IFN-γ and TNF-α production, indicating that the HPV may play a role in blocking the production of these cytokines in NK cells. The effect observed when we employed the HO-1 inhibitor on IFN-γ and TNF-α production in CD107a + NK-92 cells co-cultured with CCC was important because IFN-γ and TNF-α produced by NK cells can induce apoptosis in tumor cells by means of interaction with localized cell-death receptors in tumor cells or through stimulation of cytotoxicity activity in CD8+ cells, in addition to helping to differentiate CD4+ T cells toward a Th1 response to promote CD8+ cell differentiation and to promote antitumor antibody production by B cells [47-50]. In addition, soluble E6 and E7 oncoproteins of HPV-16 inhibit NK cells to produce IFN [51]. Activating receptors are very important in NK activity, and it was reported that tumor cells can induce a decrease in the expression of activation receptors in NK cells, the latter through several mechanisms, such as binding competitively to the activating receptor and disrupting the binding between the activating receptor and the ligand expressed by tumor cells or by inducing the internalization of the activating receptor on NK cells [52,53]. In another study, we showed that CCC can induce downmodulation of the NKG2D receptor [54]. This is important because we observed that when HeLa and SiHa cells were pre-treated with the HO-1 inhibitors (SnPP and ZnPP) and subsequently co-cultured with NK-92 cells, the downmodulated expression of NKG2D and NKp30 was restored, in comparison with NK-92 cells co-cultured with HeLa and SiHa CCC without pre-treatment with HO-1 inhibitors. This same phenomenon was observed for NKp46 in HeLa, SiHa and C-33A cells. We observed a similar response in NK cells of healthy donors (data not shown). All data strengthened the importance of HO-1 in this phenomenon because we observed these effects when we employed both HO-1 inhibitors. It is noteworthy that we also observed a significant decrease in NK-92 and primary NK cells in the expression of NKp30, NKp46, and NKG2D after 24 h of their being exposed to the culture supernatant of HeLa, SiHa, and C-33A CCC. NK cells are found in the stroma of HPV-infected Cervical intraepithelial neoplasia (CIN); however, NKp30, NKp46, and NKG2D expression is reduced in patients with pre-cancerous or cancerous HPV-induced lesions and, in addition, their cytotoxicity was reduced [51,55].

We also studied the regulation of some NK cell ligands, such as MICA and MICB, in CCC and the effect of HO-1 inhibitors on their expression. However, we did not observe any change in MICA and MICB expression when we utilized HO-1 inhibitors. MICA is high expressed in HeLa and SiHa cells, but MICB is absent. In C-33A, we found higher expression of MICB in comparison with HeLa and SiHa cells. At present, we are studying whether HO-1 inhibitors exert some effect on other NK cell ligands. As already known, cancer cells possess several mechanisms to avoid the immune response, including the production of molecules with suppressor activity on the immune response; tumor cells are able to induce a regulatory phenotype in immune cells, promoting immune evasion. The difference observed in our study among HeLa, SiHa, and C-33A cells in IFN-γ, TNF-α, NCR, and NKG2D on NK cells indicates that several mechanisms of resistance to cytotoxicity and to NK cell activity could be implicated, such as HPV type and their interactions with NK cells. Our findings indicate that HO-1 can interfere with IFN-γ and TNF-α production and that are implicated in deregulation in the expression of activation receptors such as NKG2D, NKp30, and NKp46.

## Conclusions

HO-1 inhibition in cervical cancer cells HeLa, SiHa, and C-33A induce an increase in IFN-γ and TNF-α production in CD107a + NK-92 cells and restore downmodulation of NKG2D, NKp30, and NKp46 in NK cells. These results demonstrate the complexity in the different interrelations between cancer cells and immune cells. The interactions in the tumor microenvironment should be considered before designing NK cell therapies.

## Methods

### Antibodies and reagents

The following mAbs were used in this study: mouse-anti-human-IFN-γ-PE/Cy7 [BioLegend, San Diego, CA, USA); clone:4S.B3]; mouse-anti-human-CD56-PE [BioLegend, clone:HCD56]; mouse-anti-human-CD56-APC [BioLegend, clone:HCD56]; mouse-anti-human-CD107a-FITC [BioLegend, clone:H4A3]; mouse-anti-human-granzyme B-AF647 [BioLegend, clone:GB11]; mouse-anti-human-TNF-γ-PE/Cy7 [BioLegend, clone:MAb11]; mouse-anti-human-HO1 (Abcam, Cambridge, MA, USA); goat-anti-mouse-IgG-PE (Abcam); mouse-anti-human-MICA-PE [R&D systems, Mineapolis, MN, USA; clone:159227] and mouse-anti-human-MICB-APC [R&D systems, clone:236511]. LIVE/DEAD Fixable Near-IR (Invitrogen, Life Technologies, Grand Island, NY, USA), and SYTOX Red Dead Cells Stain (Invitrogen, Life Tchnologies). Zinc and Tin-protoporphyrin IX (ZnPP and SnPP, HO-1 inhibitors) was purchased from Frontier Scientific, Inc. (Logan, UT, USA). A stock solution was prepared in DMSO and protected from light. CCC were treated with the HO-1 inhibitor (SnPP) at a final concentration of 25 μM during 48 h and with Zinc protoporphyrin (ZnPP) at a final concentration of 1 μM. Viability and MICA and MICB expression were evaluated using SYTOX and MICA and MICB antibodies, respectively. The final concentration of DMSO in the cell culture medium was <0.1%.

### Cell lines

HeLa (HPV-18+), SiHa (HPV-16+), and C-33A (HPV–) CCC were kindly provided by Dr. Boukamp (DKFZH, Heidelberg, Germany). The presence of HPV type was confirmed by the Linear array genotyping test (Roche). NK-92 cells were obtained from American Type Culture Collection (ATCC CRL-2407™; Manassas, VA, USA). Blood of healthy donors was obtained with informed consent and NK primary cells were isolated using FACSAria I cell sorting (BD Biosciences, San Jose, CA, USA). The CCC (HeLa, SiHa, and C-33A) were maintained in vitro propagated in Dulbecco’s modified Eagle’s culture medium (DMEM; GIBCO™ Invitrogen Corp., Carlsbad, CA, USA) with 10% heat-inactivated Fetal bovine serum (FBS; GIBCO™ Invitrogen Corp.), and NK-92 cells were maintained in vitro propagated in RPMI-1640 culture medium (GIBCO™ Invitrogen Corp.) with 100 U/mL recombinant human IL-2 (BioLegend) and FBS at a final concentration of 15% (GIBCO™ Invitrogen Corp.); both media were supplemented with 1X L-glutamine (at a 2-mM final concentration; GIBCO™ Invitrogen Corp.) and antibiotics (penicillin/streptomycin; GIBCO™ Invitrogen Corp.). These media will be referred to as DMEM-S and RPMI-S. Cells were incubated at 37°C in a humidified atmosphere containing 95% air and 5% CO_2_.

### Supernatant of cervical cancer cell lines

CCC HeLa, SiHa, and C-33A were grown in flasks at 80–90% confluence and harvested with trypsin. After that, 100,000 HeLa, SiHa, or C-33A cells were plated on 2 mL of DMEM-S on 6-well culture plates. Cells were incubated at 37°C in a humidified atmosphere containing 95% air and 5% CO_2_ for 5 days. Afterward, the cultured supernatant of these cell lines was collected, and NK-92 and NK primary cells were treated or not with the supernatant of HeLa, SiHa and C-33A at a final concentration of 30% of the total volume. Subsequently, the cells were incubated for 24 h in a humidified atmosphere containing 95% air and 5% CO_2_.

### Evaluation of HO-1 expression on cervical cancer cell lines

Expression of HO-1 in HeLa, SiHa, and C-33A CCC lines was detected by an indirect staining protocol using a PE-conjugated anti-mouse secondary antibody after incubation with mouse anti-HO-1 primary antibody (Abcam). At least 10,000 events were acquired using an EPICS XL-MCL™ Beckman Coulter model flow cytometer (Fullerton, CA, USA). Data were processed with FlowJo ver. X.0.7 software (Tree Star, Inc., Ashland, OR, USA) and results are reported as the % of expression or geometric Mean fluorescence intensity (MFI).

### In vitro co-culture of NK-92 cells with cervical cancer lines

NK cells were co-cultured with HeLa, SiHa, and C-33A CCC pre-treated or not with HO-1 inhibitors (SnPP or ZnPP). Briefly, HeLa, SiHa, and C-33A cells (20,000) were seeded in U-bottom 96-well plates and then were placed in contact with NK-92 cells or NK primary cells at T:E (Target [CCC]:Effector cells [NK cells]) at ratios of 1:5, 1:20, and 1:40; we used PMA + Ionomycin as positive control [40]. Then we added anti-CD107a-FITC. Subsequently, the cells were incubated for 1 h at 37°C in a humidified atmosphere containing 95% air and 5% CO_2_. Afterward, we added the protein transport inhibitors Brefeldin A (BioLegend) at a final dilution of 1/1,000 and Monensin (Sigma Chemical Co., St. Louis, MO, USA) at a final concentration of 6 μg/mL. Then, the cells were cultured for 4 h at 37°C in a humidified atmosphere containing 95% air and 5% CO_2_. Afterward, we evaluated CD107a, TNF-α, IFN-γ, granzyme B, NKp30, NKp44, NKp46, and NKG2D expression in NK cells by Flow cytometry (FC).

### Transwell assay

CCC HeLa, SiHa, and C-33A were cultured with NK-92 and NK primary cells in a dual-chamber transwell (0.4-μm micropores; Costar, Corning, NY, USA). NK cells were placed into the lower chamber and CCC were placed into the upper chamber and were cultured at 37°C for 24 h. After that, the NK cells were collected and we evaluated NCR and NKG2D expression.

### Assessment of CD107a, TNF-α, IFN-γ, and granzyme B by flow cytometry

After the co-culture, we determined CD107a, TNF-α, IFN-γ and granzyme B (BioLegend) by FC. First, NK-92 cells were stain for viability using LIVE/DEAD Fixable Near-IR. After that NK-92 cells were harvested and resuspended in PBS, stained with CD56-PE, and incubated in the dark for 30 min at room temperature. Afterward, the cells were washed and fixed with fixation buffer (BioLegend) for 15 min. Then, the cells were washed and permeabilized with permeabilization buffer 1X (BioLegend), we added TNF-γ-PE-Cy7, granzyme B-AF647, or IFN-α-PE-Cy7, and the cells were incubated in the dark for 30 min. Then, the cells were washed with PBS, fixed with paraformaldehyde 1 %, and analyzed by FC. An appropriate isotype and Fluorescence Minus One (FMO) controls were utilized to adjust for background fluorescence, and results are reported as the % of expression. For each sample, at least 10,000 events were acquired in a FACSAria I cell sorter (BD Biosciences). Data were processed with FlowJo ver. X.0.7 software (Tree Star, Inc.).

### Assessment of NKp30, NKp44, NKp46, and NKG2D by flow cytometry

After co-culture, we evaluated the expression of NKp30, NKp44, NKp46, and NKG2D in NK cells by FC. First, NK cells were stain for viability using LIVE/DEAD Fixable Near-IR, after that NK cells were harvested, resuspended in PBS, and stained with CD56-APC, NKp30-PE, NKp44-PE, NKp46-PE, or NKG2D-PE (BioLegend); subsequently, the cells were incubated in the dark for 30 min at room temperature. Cells were washed and fixed with paraformaldehyde 1%. An appropriate isotype and FMO controls were utilized to adjust for background fluorescence, and results are reported as the % of expression or geometric Mean fluorescence intensity (MFI). For each sample, at least 10,000 events were acquired in a FACSAria I cell sorter (BD Bioscience). Data were processed with FlowJo ver. X.0.7 software (Tree Star, Inc.).

### Statistical analysis

All experiments were carried out in triplicate and were repeated three times. The values represent mean ± Standard deviation (SD) of the values obtained. Statistical analysis was performed with the non-parametric Mann–Whitney *U* test, considering *p* <0.05 as significant.
